# The Mechanism of Hsp90 ATPase Stimulation by Aha1

**DOI:** 10.1038/srep33179

**Published:** 2016-09-12

**Authors:** Annemarie Wolmarans, Brian Lee, Leo Spyracopoulos, Paul LaPointe

**Affiliations:** 1 Department of Cell Biology, 514 Medical Sciences Building, University of Alberta, Edmonton, Alberta, T6G 2H7, Canada; 2 Department of Biochemistry, 416 Medical Sciences Building, University of Alberta, Edmonton, Alberta, T6G 2H7, Canada

## Abstract

Hsp90 is a dimeric molecular chaperone responsible for the folding, maturation, and activation of hundreds of substrate proteins called ‘clients’. Numerous co-chaperone proteins regulate progression through the ATP-dependent client activation cycle. The most potent stimulator of the Hsp90 ATPase activity is the co-chaperone Aha1p. Only one molecule of Aha1p is required to fully stimulate the Hsp90 dimer despite the existence of two, presumably identical, binding sites for this regulator. Using ATPase assays with Hsp90 heterodimers, we find that Aha1p stimulates ATPase activity by a three-step mechanism via the catalytic loop in the middle domain of Hsp90. Binding of the Aha1p *N* domain to the Hsp90 middle domain exerts a small stimulatory effect but also drives a separate conformational rearrangement in the Hsp90 *N* domains. This second event drives a rearrangement in the *N* domain of the opposite subunit and is required for the stimulatory action of the Aha1p *C* domain. Furthermore, the second event can be blocked by a mutation in one subunit of the Hsp90 dimer but not the other. This work provides a foundation for understanding how post-translational modifications regulate co-chaperone engagement with the Hsp90 dimer.

The 90 kiloDalton heat shock protein (Hsp90) is a molecular chaperone that plays an essential role in protein folding in cells^1^,^2^,^3^,^4^. Hsp90 regulates the folding, conformational maturation, and assembly of a large group of substrate proteins termed clients^5^,^6^,^7^,^8^,^9^,^10^,^11^,^12^,^13^. Hsp90 client proteins include kinases, hormone receptors and other transcription factors, membrane proteins, and a variety of proteins with no obvious sequence or structural similarity. Client maturation by the Hsp90 dimer occurs in the context of an ATP-driven functional cycle during which Hsp90 undergoes global conformational rearrangements that involve inter- and intra-protomer interactions^14^,^15^,^16^. Each protomer of the Hsp90 dimer is comprised of an *N* terminal ATP-binding domain, a middle domain, and a *C* terminal dimerization domain (Fig. 1A)^17^,^18^,^19^,^20^,^21^,^22^. These domains are joined by long, charged, flexible linkers that allow the dimer to undergo dramatic conformational rearrangements.

The client activation cycle is regulated by the sequential interaction of regulatory proteins called co-chaperones that recognize discrete conformational states^8^,^23^,^24^. Co-chaperone proteins guide the client maturation cycle presumably by regulating the transition between conformational states that ultimately result in ATP hydrolysis^8^,^24^,^25^. The ability to bind and hydrolyze ATP is essential for Hsp90 function *in vivo* as Hsp90 mutants that cannot bind or hydrolyze ATP do not support viability in yeast^26^,^27^. The most potent stimulator of the low ATPase activity of Hsp90 is Aha1, or the “activator of Hsp90 ATPase’’^19^,^28^,^29^,^30^. This co-chaperone has been shown to play a critical role in kinase activation and membrane protein folding in mammalian cells, however, the mechanism of Aha1 action is poorly understood^11^,^31^. Aha1 is comprised of two domains; a 156 residue *N* terminal domain and a similarly sized *C* terminal domain that are joined by an unstructured linker (Fig. 1B)^32^,^33^. ATPase stimulation is driven by two main interactions between Aha1p and Hsp90. The *N* terminal domain of Aha1p interacts with the middle domain of Hsp90 and is thought to elicit a conformational rearrangement in the Hsp90 *N* domains (Fig. 1C)^33^. The Aha1p *C* terminal domain interacts with the dimerized *N* terminal domains of the Hsp90 dimer (Fig. 1C)^32^,^33^. The relative contributions of these two interactions to ATPase stimulation or the underlying mechanics are not understood.

Yeast possess a co-chaperone called Hch1p that is homologous to the Aha1p *N* terminal domain (Aha1p^N^) (Fig. 1B), which is useful for interrogating domain rearrangements that occur upon interaction with the middle domain^19^,^28^,^29^,^34^. We have shown that Hch1p, but not Aha1p, overexpression in yeast increases the cellular sensitivity to specific, ATP-competitive Hsp90 inhibitors like NVP-AUY922^28^. Furthermore, Hch1p interacts genetically with Hsp90 alleles that are not affected by Aha1p expression. Despite the differences in the biology of these two co-chaperones, both Hch1p and Aha1p^N^ can stimulate the ATPase activity of Hsp90 to a similar degree^19^,^28^,^29^. Interestingly, though Hch1p has evolved to function as a single domain *in vivo*, fusion of Aha1p^C^ to Hch1p enhances ATPase stimulation. ATPase stimulation by this Hch1p-Aha1p chimera (Fig. 1B) is lower than that achieved by full length Aha1p but consistent with both Hch1p and Aha1p^N^ facilitating the activity of the Aha1p *C* domain. However, we have shown that a mutation in the catalytic loop of Hsp90, E381K, impairs ATPase stimulation by Aha1p and Aha1p^N^ but not by Hch1p or the chimera^29^. Importantly, the catalytic loop (residues 370–390 in the Hsp90 middle domain) mediates communication with the *N* domain of Hsp90^30^. Binding of Aha1p^N^ to the middle domain drives a conformational change in the catalytic loop that remodels residues in the ATP binding pocket. Thus, the Aha1p *N* domain has likely evolved to specifically facilitate the action of the Aha1p *C* domain by manipulating the catalytic loop in a way that Hch1p has not. Thus, comparing Hch1p and Aha1p can provide biological insight into the mechanics of Hsp90 regulation.

ATP hydrolysis by Hsp90 is intricately choreographed involving collaboration between numerous structural elements in the dimer structure^20^,^22^,^35^. Hsp90 dimerization is obligatory for function, and maintained by interaction between *C* terminal domains. Dimerization of the *N* terminal domains is required, albeit not sufficient, for ATP hydrolysis. Deletion of the *N* terminal ‘strap’ (residues 1–24) in one subunit of an Hsp90 dimer eliminates ATPase activity in the opposite protomer, demonstrating the importance of interdomain contact between the *N* domains^35^,^36^. Additionally, the ATPase activity of Hsp90 is auto-inhibited by a ‘lid’ that closes over bound nucleotide^37^. Deletion of the lid segment in both subunits of an Hsp90 dimer eliminates ATPase activity, however, deletion of this segment in only one subunit dramatically enhances ATPase activity in the opposite subunit in the absence of co-chaperones^37^,^38^. Moreover, progression through at least five different conformational states is accelerated in a similar manner upon deletion of the lid segment in one subunit or the addition of Aha1p, suggesting that this may be the mechanism by which Aha1p stimulates ATPase activity^38^.

In this report, we have studied the nature of asymmetric ATPase stimulation of Hsp90 by the intact Aha1p co-chaperone, and how the *N* terminal orthologue Hch1p differs from Aha1p^N^ in this regard. Our work defines three discrete steps in Aha1p-mediated ATPase stimulation of the Hsp90 dimer and provides mechanistic insight into the different activities of Hch1p and Aha1p. In addition, we have found that the *C*-terminal domain of Aha1p is required for ‘co-chaperone switching’ *in vitro*.

## Results

### Regulation of Hsp90 lid dynamics by Aha1p and Hch1p

**Figure 2:**
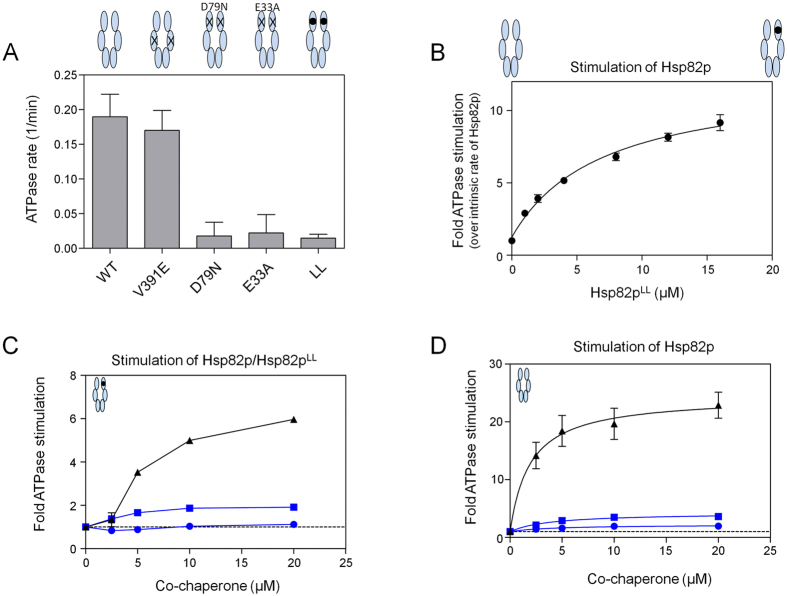
ATPase stimulation of Hsp82p:Hsp82p^LL^ heterodimers. (**A**) Bar graph showing the intrinsic ATPase rates of wildtype Hsp82p and Hsp82p mutants (Hsp82p^V391E^, Hsp82p^D79N^, Hsp82p^E33A^, and Hsp82p^LL^). Reactions contained 4 μM of Hsp82p or Hsp82p mutants. ATPase rate shown in micromolar ATP hydrolyzed per minute per micromolar of enzyme (1/min). (**B**) The addition of Hsp82p^LL^ potently stimulates the ATPase activity of wildtype Hsp82p. Hsp82p^LL^ was titrated into reactions containing 2 μM of wildtype Hsp82p. The resulting stimulated ATPase rate is shown as a fold stimulation of the intrinsic Hsp82p rate. (**C**) Titration of Aha1p and Aha1p^N^ stimulates the ATPase activity of Hsp82p:Hsp82p^LL^ heterodimers. Titration of Hch1p inhibits the ATPase activity of Hsp82p:Hsp82p^LL^ heterodimers at low concentrations (by ~16% at 2.5 μM Hch1p and ~11% at 5 μM Hch1p). Heterodimers are formed by mixing 1 μM Hsp82p and 5 μM of Hsp82^LL^. ATPase rates are shown as a fold stimulation the intrinsic rate of Hsp82p:Hsp82p^LL^ heterodimers (stippled line). (**D**) ATPase stimulation of wildtype Hsp82p by Aha1p, Hch1p and Aha1p^N^. The V_max_ values for co-chaperone stimulation by each of these co-chaperones are 3.8 ± 0.4 min^−1^ (Aha1p), 0.4 ± 0.1 min^−1^ (Hch1p), and 0.8 ± 0.1 min^−1^. Reactions contained 2 μM of Hsp82p with indicated concentrations of co-chaperones. All Aha1p, Hch1p, and Aha1p^N^ titrations are shown as black triangles, blue circles, and blue squares, respectively. The ATPase rate is shown as a fold stimulation of the intrinsic Hsp82p rate (stippled line).

The ATPase activity of Hsp90 is auto-inhibited by a ‘lid’ segment (residues 98–121 in yeast Hsp82p) that functions as a gate to regulate nucleotide binding and commitment to hydrolysis^37^. The dramatic enhancement in ATPase activity that occurs upon deletion of this lid in one subunit of an Hsp90 dimer implies that efficiency of ATP hydrolysis is regulated by lid position. Interestingly, the progression through five different conformational states associated with the ATPase cycle, is similarly altered upon addition of Aha1p, or deletion of the lid segment in one protomer of Hsp90 in the context of a heterodimer consisting of one wildtype subunit and one lidless subunit (Hsp82p^LL^)^38^. A straightforward explanation is that Aha1p promotes lid opening, driving *N* domain dimerization, and acceleration of ATP hydrolysis. In this case, Aha1p would be unable to stimulate an Hsp82p:Hsp82p^LL^ heterodimer which has enhanced ATPase activity compared to a wildtype homodimer. Consistent with previous reports, the addition of Hsp82p^LL^, which like Hsp82p^D79N^ and Hsp82p^E33A^, lacks ATPase activity (Fig. 2A), potently stimulates the ATPase activity of wildtype Hsp82p (Fig. 2B)^37^. However, titration of Aha1p into reactions containing Hsp82p:Hsp82p^LL^ heterodimers indicates that Aha1p further stimulates ATPase activity (Fig. 2C). As is the case with stimulation of wildtype Hsp82p, Aha1p^N^ was able to stimulate the ATPase activity of Hsp82p:Hsp82p^LL^ heterodimers but to a lesser degree than full length Aha1p (Fig. 2C). This suggests that while the lidless-stimulated Hsp82p:Hsp82^LL^ heterodimer relieves auto-inhibition of ATP hydrolysis to some extent, the *N* domain of Aha1p is important in subsequent remodelling of the Hsp90 *N* domains to facilitate catalysis.

Given the different biological activities of Hch1p and Aha1p^28^,^29^, it is of interest to probe the stimulatory mechanism of Hch1p, which shares 37% amino acid identity with the Aha1p *N* domain^28^. Unexpectedly, we found that Hch1p inhibits the ATPase activity of Hsp82p:Hsp82p^LL^ heterodimers in a manner that is gradually overcome at higher co-chaperone concentrations (Fig. 2C). This suggested to us that the ATPase activation mechanism of Hch1p differs from that of Aha1p^N^. In principle, Hch1p can bind to either the ATPase-competent subunit, the lidless subunit, or both in these experiments. We observed the greatest inhibition (~16% of the intrinsic rate of the Hsp82p:Hsp82p^LL^ heterodimer) when 2.5 μM Hch1p was added to the reaction. When the Hch1p concentration reached 5 μM, ATPase activity was only inhibited by ~11%. Since the total amount of Hsp82p was 6 μM, inhibition was likely due to binding to one subunit of the heterodimer. ATPase stimulation of wildtype Hsp82p homodimers by each of these co-chaperones is shown for comparison (Fig. 2D). The maximal ATPase rates we observed when co-chaperones were added to wildtype Hsp82p were 3.8 ± 0.4 min^−1^ for Aha1p, 0.4 ± 0.1 min^−1^ for Hch1p, and 0.8 ± 0.1 min^−1^ for Aha1p^N^. Since Hch1p could bind to either subunit of Hsp82p:Hsp82p^LL^ heterodimers we pursued a strategy to restrict co-chaperone binding to one subunit or the other.

### Aha-type co-chaperones exert different effects from different subunits of Hsp90 heterodimers

**Figure 3:**
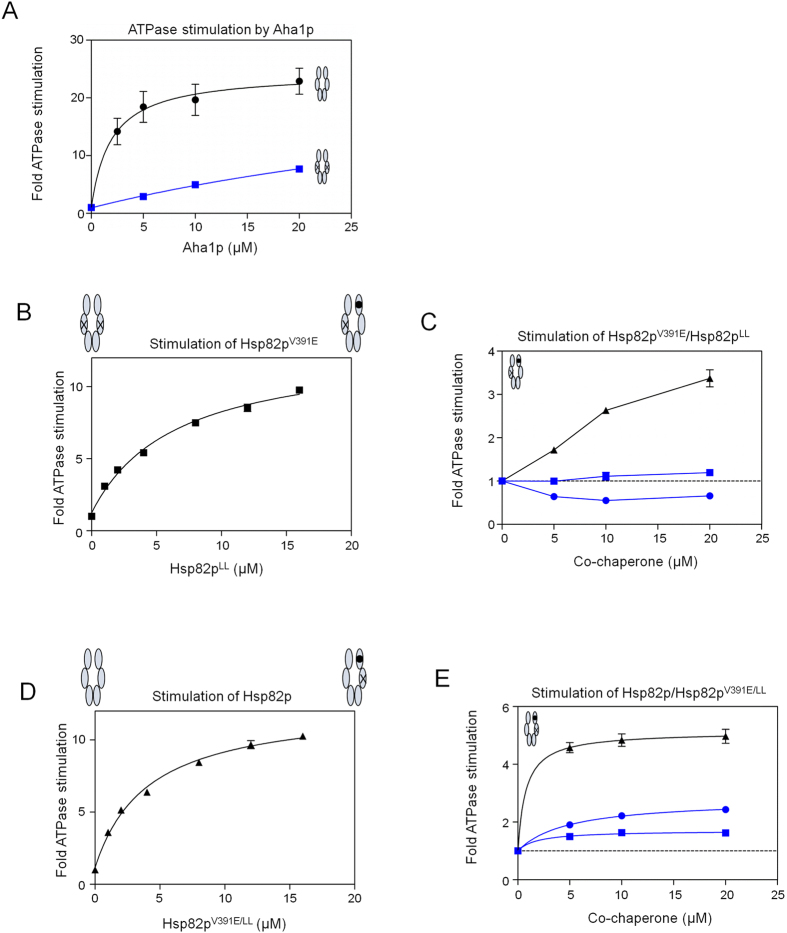
Co-chaperone action is protomer specific; Co-chaperones stimulate the ATPase activity of Hsp82p when co-chaperone binding is restricted to the catalytic protomer, but not the non-catalytic protomer. (**A**) Aha1p stimulates wildtype Hsp82p (black) robustly but does not readily stimulate Hsp82p^V391E^ (blue). Reactions contained 2 μM Hsp82p or Hsp82p^V391E^ with indicated Aha1p concentrations. ATPase rate shown as a fold stimulation of Hsp82p or Hsp82p^V391E^ intrinsic rate. (**B**) The addition of Hsp82p^LL^ potently stimulates the ATPase activity of Hsp82p^V391E^. Hsp82p^LL^ was titrated into reactions containing 2 μM of Hsp82p^V391E^. The resulting stimulated ATPase rate is shown as a fold stimulation of the intrinsic Hsp82p^V391E^ rate. (**C**) Aha1p (black triangles) and Aha1p^N^ (blue squares) do not robustly stimulate the ATPase activity of Hsp82p^V391E^:Hsp82p^LL^ heterodimers, and Hch1p (blue circles) inhibited the ATPase activity of Hsp82p^V391E^:Hsp82p^LL^ heterodimers. Heterodimers are formed by mixing 1 μM Hsp82p^V391E^ and 5 μM Hsp82p^LL^. ATPase rates are shown as a fold stimulation of the intrinsic rate of Hsp82p^V391E^:Hsp82p^LL^ heterodimers (stippled line). (**D**) The addition of Hsp82p^V391E/LL^ potently stimulates the ATPase activity of wildtype Hsp82p. Hsp82p^V391E/LL^ was titrated into reactions containing 2 μM of Hsp82p. The resulting stimulated ATPase rate is shown as a fold stimulation of the intrinsic Hsp82p rate. (**E**) Aha1p (black triangles), Hch1p (blue circles), and Aha1p^N^ (blue squares) stimulates the ATPase activity of Hsp82p:Hsp82p^V391E/LL^ heterodimers. Heterodimers are formed by mixing 1 μM Hsp82p and 5 μM of Hsp82^V391E/LL^. ATPase rates are shown as a fold stimulation the intrinsic rate of Hsp82p:Hsp82p^V391E/LL^ heterodimers (stippled line).

To determine how Hch1p influences ATPase activity from intact or lidless subunits, we employed the V391E mutation to diminish co-chaperone binding to the middle domain of Hsp82p^33^. We first confirmed that Hsp82p^V391E^ homodimers have normal intrinsic ATPase activity (Fig. 2A) but are not readily stimulated by Aha1p (Fig. 3A). We also verified that Hsp82p^V391E^ ATPase activity is stimulated by the addition of Hsp82p^LL^ (Fig. 3B). In Hsp82p^V391E^:Hsp82p^LL^ heterodimers, co-chaperone binding will occur preferentially to the lidless protomer^37^. Aha1p and Aha1p^N^ were less effective in stimulating the ATPase activity of Hsp82p^V391E^:Hsp82p^LL^ heterodimers (Fig. 3C) than Hsp82p:Hsp82p^LL^ heterodimers (compare to Fig. 2C). It is important to note that the V391E mutation does not block co-chaperone binding completely, but rather, reduces the affinity by roughly 10 fold^33^. Thus, the weak stimulation that we observe is likely due to weak binding to the Hsp82p^V391E^ subunit of the heterodimer. These data suggest that neither Aha1p nor Aha1p^N^ can stimulate ATPase activity in the Hsp82p^V391E^ subunit when bound to the Hsp82p^LL^ subunit. Furthermore, Hch1p was only inhibitory in these ATPase assays suggesting that binding to the Hsp82p^LL^ protomer actually antagonizes the enzymatic activity of the Hsp82p^V391E^ subunit of these heterodimers. We next tested Hsp82p:Hsp82p^V391E/LL^ heterodimers for stimulation by our co-chaperone constructs. In this case, co-chaperones will preferentially bind to the wildtype subunit. As with our other lidless heterodimers, addition of Hsp82p^V391E/LL^ stimulates ATPase activity in wildtype Hsp82p (Fig. 3D). In contrast to Hsp82p^V391E^:Hsp82p^LL^ heterodimers, each of Aha1p, Aha1p^N^, and Hch1p stimulated the ATPase activity of Hsp82p:Hsp82p^V391E/LL^ heterodimers (Fig. 3E).

These data may suggest that Aha1p enhances the conformational changes in the Hsp90 dimer that are driven by deletion of the lid segment. Alternatively, Aha1p interaction and lid deletion may cause discrete conformational changes in the Hsp90 dimer.

### ATPase stimulation of Hsp90 ATP hydrolysis mutant heterodimers by Aha1p and Hch1p

**Figure 4:**
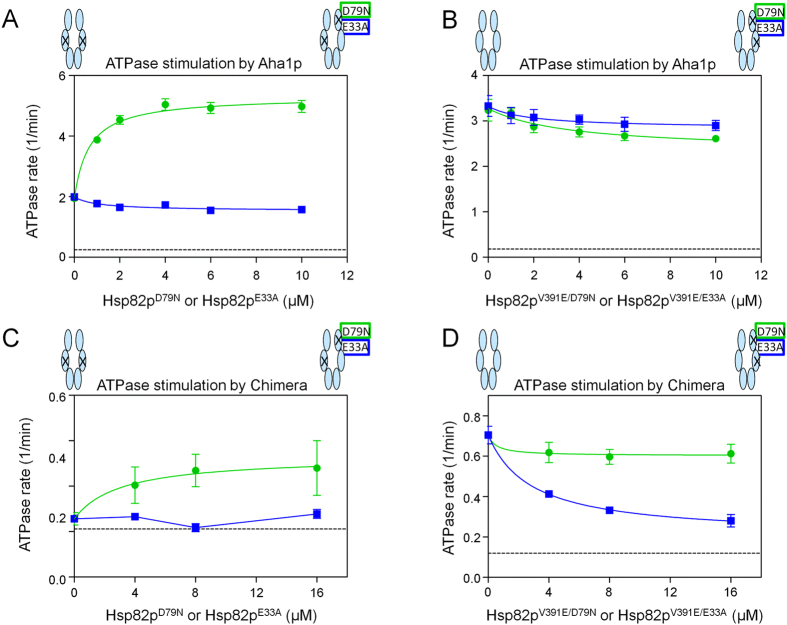
The E33A mutation blocks Aha1p^N^-mediated conformational changes in *cis*. (**A**) Aha1p-mediated ATPase stimulation of Hsp82p^V391E^ was restored in trans with Hsp82p^D79N^ (green) but not Hsp82p^E33A^ (blue). Reactions contained 1 μM Hsp82p^V391E^, 10 μM Aha1p and indicated concentrations of Hsp82p^D79N^ or Hsp82p^E33A^. Intrinsic rate of Hsp82p^V391E^ shown as black stippled line. (**B**) Aha1p-mediated ATPase stimulation of Hsp82p:Hsp82p^V391E/D79N^ (green) and Hsp82p:Hsp82p^V391E/E33A^ (blue) heterodimers. Reactions contained 1 μM Hsp82p, 10 μM Aha1p and indicated concentrations of Hsp82p^V391E/D79N^ or Hsp82p^V391E/E33A^. Intrinsic rate of wildtype Hsp82p shown as black stippled line. (**C**) Chimera-mediated ATPase stimulation of Hsp82p^V391E^ was restored in trans with Hsp82p^D79N^ (green) but not Hsp82p^E33A^ (blue). Reactions contained 1 μM Hsp82p^V391E^, 10 μM Chimera, and indicated concentrations of Hsp82p^D79N^ or Hsp82p^E33A^. Intrinsic rate of Hsp82p^V391E^ shown as black stippled line. (**D**) Chimera-mediated ATPase stimulation of Hsp82p:Hsp82p^V391E/D79N^ (green) heterodimers but not of Hsp82p:Hsp82p^V391E/E33A^ (blue) heterodimers. Reactions contained 1μM Hsp82p, 10 μM Chimera and indicated concentrations of Hsp82p^V391E/D79N^ or Hsp82p^V391E/E33A^. Intrinsic rate of wildtype Hsp82p shown as black stippled line. All ATPase rates are shown in micromolar ATP hydrolyzed per minute per micromolar of enzyme (1/min).

In order to further probe conformational remodelling and ATPase stimulation by Aha1p and Hch1p, we used ATP binding deficient (D79N) and ATP hydrolysis deficient (E33A) Hsp82p mutants that are otherwise structurally intact^26^,^27^,^33^,^37^. A study of engineered Hsp82p heterodimers found that Hsp82p:Hsp82p^E33A^ heterodimers support yeast viability whereas Hsp82p:Hsp82p^D79N^ heterodimers do not^39^. To explore the nature of catalytic activity for Hsp82p heterodimers where only one subunit can hydrolyze ATP, we added either Hsp82p^D79N^ or Hsp82p^E33A^ (which can each bind Aha1p but lack ATPase activity) to ATPase reactions containing Aha1p and Hsp82p^V391E^ (which can hydrolyze ATP but not bind Aha1p). Consistent with previous reports, we find that addition of Hsp82p^D79N^ restores Aha1p-mediated ATPase stimulation of Hsp82p^V391E^ (Fig. 4A)^33^. Intriguingly, addition of Hsp82p^E33A^ does not restore Aha1p-mediated ATPase stimulation to Hsp82p^V391E^ (Fig. 4A). This demonstrates that Aha1p can stimulate the ATPase activity of the ATPase-competent Hsp82p^V391E^ subunit when it is bound to an Hsp82p^D79N^, but not an Hsp82p^E33A^ subunit of a heterodimer. In other words, the E33A mutation ablates the ability of Aha1p to act asymmetrically from the ATPase-dead subunit. Importantly, neither the addition of Hsp82p^V391E/D79N^ nor Hsp82p^V391E/E33A^ (which cannot bind Aha1p or hydrolyze ATP) diminished Aha1p-stimulated ATPase activity of wildtype Hsp82p (Fig. 4B). This indicates that binding of Aha1p to the ATPase-competent subunit of a heterodimer can stimulate ATP hydrolysis regardless of whether the D79N or E33A mutation is present in the opposite subunit. Importantly, we confirmed that Hsp82p:Hsp82p^E33A^ and Hsp82p:Hsp82p^D79N^ heterodimers form equally well (Figure S1) and that Aha1p interacted with each of our Hsp82p mutants except Hsp82p^V391E^ (Figure S2).

**Figure 5:**
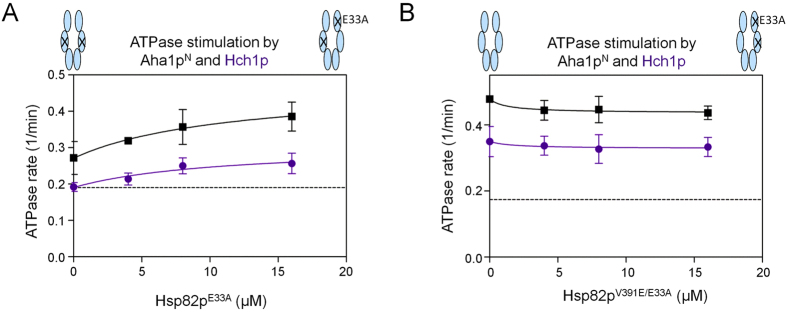
*N*-terminal Aha1p-type co-chaperones stimulate Hsp82p ATPase activity from either catalytic or non-catalytic protomer. (**A**) Hch1p- and Aha1p^N^-mediated ATPase stimulation of Hsp82p^V391E^:Hsp82p^E33A^ heterodimers. Reactions contained 4 μM Hsp82p^V391E^, 20 μM Hch1p (purple) or Aha1p^N^ (black), and indicated concentrations of Hsp82p^E33A^. Intrinsic rate of Hsp82p^V391E^ shown as black stippled line. (**B**) Hch1p- and Aha1p^N^-mediated ATPase stimulation of Hsp82p:Hsp82p^V391E/E33A^ heterodimers. Reactions contained 4 μM Hsp82p, 20 μM Hch1p (purple) or Aha1p^N^ (black), and indicated concentrations of Hsp82p^V391E/E33A^. Intrinsic rate of wildtype Hsp82p shown as black stippled line. ATPase rates are shown in micromolar ATP hydrolyzed per minute per micromolar of enzyme (1/min).

The importance of direct contact between the Aha1p *N* terminal domain and the middle domain of the catalytically active protomer of the Hsp90 dimer for ATPase stimulation by intact Aha1p raises the question of whether Hch1p can fulfill this role. We tested the Hch1p-Aha1p chimera for the ability to stimulate Hsp82p heterodimers. Similar to the results with Aha1p, chimera-mediated stimulation of Hsp82p^V391E^ is restored with addition of Hsp82p^D79N^ but not Hsp82p^E33A^ (Fig. 4C). However, while the addition of Hsp82p^V391E/D79N^ does not affect chimera-mediated ATPase stimulation of wildtype Hsp82p (Fig. 4D), the addition of Hsp82p^V391E/E33A^ greatly diminishes stimulation (Fig. 4D). This suggests that the chimera cannot stimulate Hsp82p:Hsp82p^E33A^ heterodimers from either subunit, perhaps because Hch1p cannot stimulate these heterodimers at all. To address this, we tested the Aha1p *N* domain and Hch1p for the ability to stimulate Hsp82p:Hsp82p^E33A^ heterodimers. Interestingly, the addition of Hsp82p^E33A^ promotes ATPase stimulation of Hsp82p^V391E^ by both Aha1p^N^ and Hch1p (Fig. 5A). Moreover, addition of Hsp82p^V391E/E33A^ does not impair ATPase stimulation of Hsp82p by either co-chaperone construct (Fig. 5B). This suggests that the Aha1p *C* domain portion of the chimera is unable to participate in the ATPase stimulation of Hsp82p:Hsp82p^E33A^ heterodimers. Moreover, the action of the Aha1p *C* domain in the stimulation of a heterodimer harbouring a single E33A mutation can only be restored by the interaction of the Aha1p *N* domain with the middle domain of the opposite subunit.

### Hch1p interaction with the middle domain of Hsp90 drives N-M communication

We have previously shown that overexpression in yeast of Hch1p, but not Aha1p, confers hypersensitivity to ATP-competitive inhibitors of Hsp90^28^. We have also shown that a mutation in the middle domain of Hsp82p (*i.e.* E381K in the catalytic loop) impairs Aha1p-mediated ATPase stimulation but not ATPase stimulation by Hch1p^29^. Taken together with the data described above, it is clear that Aha1p and Hch1p regulate Hsp90 differently. Given the paucity of available structures of Aha1p with intact Hsp90, we employed an NMR strategy to examine conformational changes that occur in Hsp90 upon co-chaperone interaction. To investigate the difference between Aha1p and Hch1p interaction with Hsp90, we added Aha1p^N^ or Hch1p, with or without ATP, to the N-M fragment of Hsp82p (Hsp82p^N-M^) and examined chemical shift changes in the Hsp82p^N-M^ spectra. As the affinities of ATP and the co-chaperones for Hsp82p are in the μM range^40^,^41^, the interaction is likely in the intermediate/slow-exchange regime on the NMR timescale. Titration of ligand would therefore result in a gradual appearance of peaks at their bound-state position, rather than a gradual movement towards that position. As such, the bound-state peaks cannot be easily assigned to particular residues. Thus, we adopted the approach in which the bound-state peaks were assigned to the same residue as the closest free-state peak^40^.

**Figure 6:**
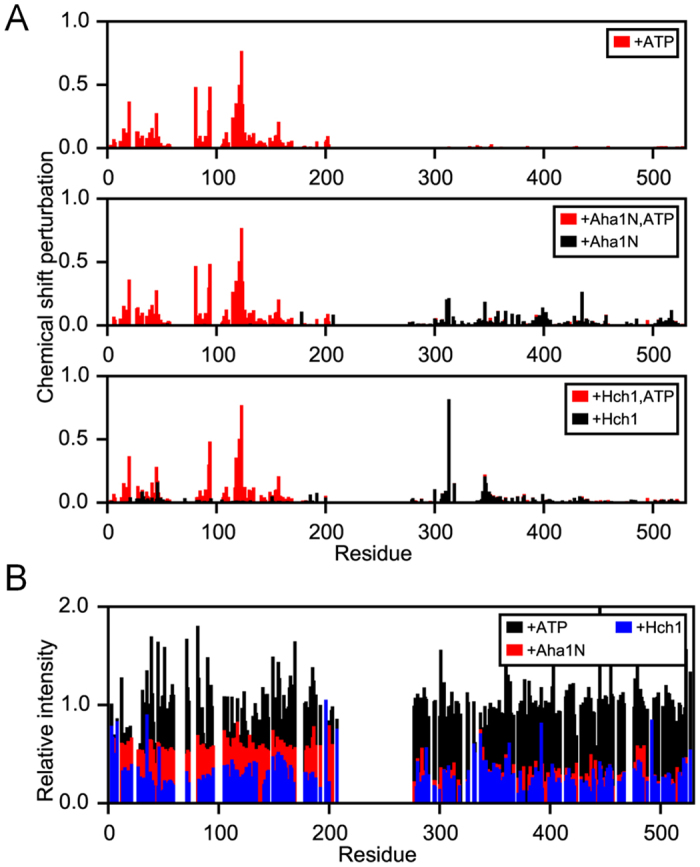
Chemical shift and peak intensity analysis in an Hsp82p N-M construct upon ATP, and co-chaperone binding. (**A**) Chemical shift changes for Hsp82p N-M construct upon addition of ATP (red, top panel), Aha1p^N^ (black, middle panel), Aha1p^N^ and ATP (red, middle panel), Hch1p (black, lower panel), or Hch1p and ATP (red, lower panel). (**B**) Peak intensity changes in Hsp82p N-M construct NMR spectra upon addition of ATP (black), Aha1p^N^ (red), or Hch1p (blue).

Chemical shift changes are observed upon addition of ATP (Fig. 6A - top panel), Aha1p^N^ with or without ATP (Fig. 6A - middle panel), or Hch1p with or without ATP (Fig. 6A - bottom panel). The addition of near saturating concentrations of ATP causes large shifts in peaks corresponding to the Hsp82p *N* domain, consistent with those described previously^40^. Aha1p^N^-driven chemical shift changes induced in Hsp82p^N-M^ are also consistent with previous studies^30^, localized primarily to the middle domain and the *C* terminal end of the *N* domain. The addition of ATP to Aha1p^N^-bound Hsp82p^N-M^ causes chemical shift changes within the *N* domain identical to those for ATP alone. The addition of Hch1p to Hsp82p^N-M^ results in a similar pattern of chemical shift changes to the middle domain, suggesting it binds in a similar manner to Aha1p^N^. However, in contrast to Aha1p^N^, Hch1p also causes changes in residues ~30–50 of the *N* domain, suggesting that Hch1 either interacts with this region or indirectly affects the conformation of the *N* domain. Adding ATP to Hch1p-bound Hsp82p^N-M^ resulted in changes in the *N* domain only, similar to the addition of ATP alone and ATP with Aha1p^N^.

The interaction, or conformational change in the *N* domain of Hsp82p^N-M^ upon addition of Hch1p is also supported by changes in peak intensity for the NMR spectra upon addition of the various ligands. Lower intensity peaks represent more slowly tumbling, larger molecular-weight species. The normalized intensities of the peaks between free-state Hsp82p^N-M^ and ATP, Aha1p^N^ and Hch1p-bound Hsp82p^N-M^ are shown in Fig. 6B. The addition of ATP does not noticeably change the intensities of the peaks, suggesting that there is little change in the overall conformation of the Hsp82p^N-M^ construct. The addition of Aha1p^N^ causes an overall decrease in peak intensity with a larger decrease in the intensities of the M domain peaks, consistent with Aha1p^N^ binding to the M domain and only weak interaction between the *N* and M domains. Addition of Hch1p results in a decrease in intensity of all of the peaks in Hsp82p^N-M^ to a similar degree. Consistent with the chemical shift data, this suggests that Hch1p binding results in a strong interaction of the *N* domain with either Hch1p or the M domain. The addition of full-length Aha1p to Hsp82p^N-M^ resulted in a pronounced broadening of many peaks making assignment difficult, particularly in the M domain. However, this is not surprising given the size of the complex between the N-M construct and full length Aha1p. The large decrease in peak intensities in the *N* domain (Figure S3), along with chemical shifts similar to those described previously^33^, show a stronger interaction between the full-length Aha1p and Hsp82p^N-M^ compared to Aha1p^N^. Overall, the data suggests that the Aha1 *N* terminal domain binds to the M domain of Hsp82p and only weakly interacts with the Hsp82p *N* domain, and that the Aha1 *C* terminal domain interacts with and restricts the motion of the Hsp82p *N* domain.

Examination of the NMR data shows that peak shifts within the Hsp82p middle domain are not the same upon Hch1p or Aha1p^N^ interaction, which is consistent with their different biological activities^28^,^29^. The peaks for nineteen residues within the Hsp82p M domain shift by more than one standard deviation (1 σ) from the average shift upon Aha1p^N^ interaction whereas ten residues shift to this extent upon Hch1p binding. Moreover, only two residue-specific chemical shift changes in the M domain are shared between the two co-chaperones. Considering only Hsp82p *N* domain NMR spectra, the chemical shift changes are striking. Only two peaks from the Hsp82p N domain shift by >1 σ upon Aha1p^N^ interaction; K178 and L207. It is noteworthy that the largest shift (>2 σ) occurs for K178, the site of SUMOylation that recruits Aha1p to the Hsp90 dimer^42^. In contrast, twelve different peaks in the Hsp82p *N* domain shift by >1 σ upon Hch1p binding to the N-M construct. These residues localize to two different regions; the face of the Hsp82p *N* domain that is oriented towards the middle domain, and the nucleotide binding pocket. The former chemical shift changes suggest that Hch1p interacts directly with the Hsp82p *N* domain and the latter changes suggest that Hch1p binding influences the nucleotide-binding pocket in a way that Aha1p cannot. This is consistent with our previous work, where we have shown that Hch1p regulates the ability of Hsp90 inhibitors to bind *in vivo*^28^,^29^.

### The *C* terminal domain of Aha1p is required for ‘co-chaperone switching’

**Figure 7:**
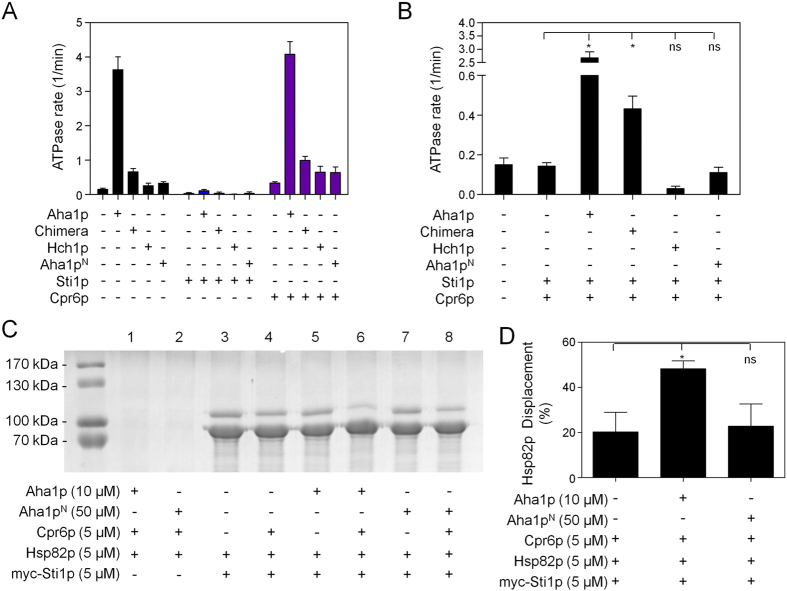
The *C* terminal domain of Aha1p is required for the cooperative displacement of Sti1p from Hsp82p. (**A**) Each co-chaperone construct (Aha1p, Chimera, Hch1p, and Aha1p^N^) stimulated Hsp82p ATPase activity (black bars). Addition of Sti1p inhibited both intrinsic and stimulated Hsp82p ATPase activity (blue bars). Addition of Cpr6p enhanced both intrinsic and stimulated Hsp82p ATPase activity (purple bars). (**B**) Only the addition of Aha1p and the Chimera to reactions containing both Sti1p and Cpr6p resulted in a statistically significant (* - one-way ANOVA) increase in ATPase activity compared to the Sti1p plus Cpr6p condition while the addition of Hch1p or Aha1p^N^ did not (n = 4). ATPase reactions (in A and B) contained 2 μM Hsp82p and 4 μM co-chaperones. ATPase rates are shown in micromolar ATP hydrolyzed per minute per micromolar of enzyme (1/min). (**C**) Co-immunoprecipitation of Hsp82p with myc-Sti1p in the presence of different combinations of co-chaperones. (**D**) Quantification of Hsp82p displacement from myc-Sti1p (n = 3). A statistically significant (* - one-way ANOVA) displacement of Hsp82p compared to the addition of Cpr6p alone was observed upon the addition of Aha1p but not a 5-fold excess of Aha1p^N^.

The Hsp90 functional cycle is thought to involve the progression through numerous co-chaperone-bound states^43^. How late-acting co-chaperones like Aha1p displace early-acting co-chaperones like Sti1p is only beginning to be revealed^43^,^44^. Sti1p is a potent inhibitor of Hsp90 ATPase activity but can be displaced by the cooperative action of Aha1p and the TPR-containing co-chaperone Cpr6p^44^. However, the mechanics of how this happens are not fully understood. Sti1p and Cpr6p bind to the MEEVD motif at the *C* terminus of each subunit of the Hsp90 dimer. Consistent with the presence of two MEEVD motifs per dimer, these two co-chaperones can exist in a ternary complex with Hsp90^44^. We wondered how each domain of Aha1p might contribute to displacement of Sti1p from the Cpr6p-Hsp90-Sti1p ternary complex to restore ATPase activity. We hypothesized that the Aha1p *C* domain is required for this ‘co-chaperone switching’. We tested Aha1p, Aha1p^N^, Hch1p, and the Hch1p-Aha1p chimera for the ability to stimulate the ATPase activity of the Cpr6p-Hsp90-Sti1p ternary complex. Consistent with previous reports we observed almost complete inhibition of Hsp90 ATPase activity by Sti1p regardless of the presence of Aha1p or Hch1p co-chaperone constructs (Fig. 7A). We also observed that Cpr6p had a mild stimulatory effect on the intrinsic Hsp90 ATPase rate as well as the stimulated rates mediated by our Aha1p and Hch1p co-chaperone constructs (Fig. 7A). These data show that ATPase stimulation mediated by all of our Aha1p and Hch1p constructs are overcome by Sti1p and augmented by Cpr6p. If the Aha1p *C* domain is required for co-chaperone switching (*i.e.* the displacement of Sti1p in the presence of Cpr6p) then only full length Aha1p and the Hch1p-Aha1p chimera would be able to stimulate the ATPase rate of reactions containing both Cpr6p and Sti1p. Consistent with this hypothesis we observed a statistically significant increase in ATPase activity in reactions containing both Sti1p and Cpr6p upon the addition of Aha1p or the Hch1p-Aha1p chimera (Fig. 7B). However, when Hch1p or Aha1p^N^ was added to reactions containing both Cpr6p and Sti1p, ATPase activity actually decreased, albeit not to a significant degree (Fig. 7B). This suggested to us that neither Hch1p nor Aha1p^N^ were able to displace Sti1p and stimulate ATPase activity. To further investigate this we employed a more direct measure of physical displacement of Hsp90 from Sti1p. We expressed and purified an *N*-terminally myc-tagged version of Sti1p for co-immunoprecipitation experiments. We incubated equimolar amounts of Hsp82p or myc-tagged Sti1p (5 μM final) together in order to co-immunoprecipitate Hsp82p with Sti1p. Hsp82p is readily recovered in complex with myc-tagged Sti1p (lane 3 - Fig. 7C). Addition of equimolar amounts of Cpr6p (5 μM) resulted in a small decrease (~20% of the total - Fig. 7D) in Hsp82p co-immunoprecipitated with myc-Sti1p (lane 4 - Fig. 7C). This is consistent with previous studies showing that Cpr6p and Sti1p can form a ternary complex with Hsp90 but also that these two co-chaperones compete for binding to the MEEVD motif at the *C* terminus of Hsp82p^44^. In contrast, addition of Aha1p (10 μM final) resulted in a negligible displacement of Hsp82p from myc-Sti1p (lane 5 - Fig. 7C). However, the addition of both Cpr6p (5 μM final) and Aha1p (10 μM final) displaced approximately 50% of Hsp82p from myc-Sti1p (lane 6 - Fig. 7C,D). Consistent with our ATPase data, the addition of a large excess (50 μM final) of Aha1p^N^ did not result in displacement of Hsp82p from myc-Sti1p on its own (lane 7 - Fig. 7C). Furthermore, addition of Aha1p^N^ together with Cpr6p did not result in any further displacement of Hsp82p compared to Cpr6p alone (lane 8 - Fig. 7C,D). Taken together, these data show that it is the *C* terminal domain of Aha1p that is required for the displacement of Sti1p from Hsp82p in cooperation with Cpr6p.

## Discussion

**Figure 8:**
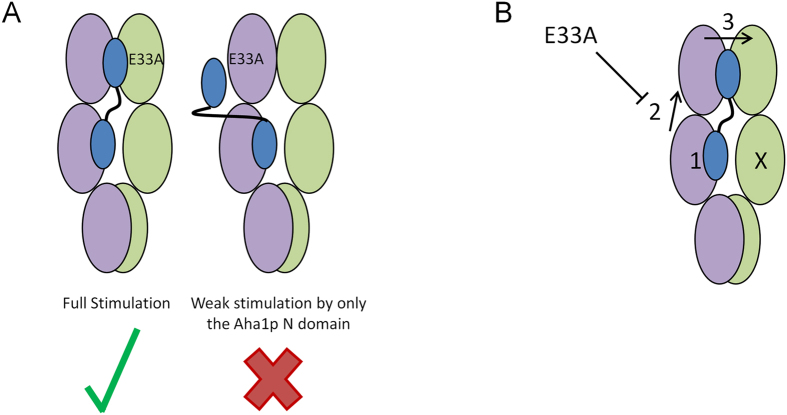
Three-step model for Aha1p-mediated stimulation of Hsp90 ATPase activity. (**A**) The E33A mutation blocks ATPase stimulation by the Aha1p *C* terminal domain only in *cis* to the Aha1p *N* domain interaction. (**B**) The first step occurs when the Aha1p *N* terminal domain interacts with the Hsp82p middle domain, driving a small increase in ATPase activity (1). The second step is the *cis* rearrangement of the Hsp82p *N* domain, likely via interaction with the catalytic loop (2). The third step is a final rearrangement of one or both Hsp82p *N* domains that allows for the participation of the Aha1p *C* domain in full ATPase stimulation (3).

The observation that Aha1p can stimulate the ATPase activity of an Hsp90 dimer from either subunit defines a model for the asymmetric action of Aha1p but does not provide mechanistic insight into how Aha1p drives the process^33^. We report here that the E33A mutation blocks the ability of Aha1p to stimulate ATP hydrolysis in the opposite subunit of a heterodimer. However, Aha1p^N^ can exert a small stimulatory effect from either subunit of a heterodimer harbouring one Hsp82p^E33A^ subunit. This suggests that the E33A mutation blocks the rearrangement of the Hsp90 *N* domains that is required for the action of the Aha1p *C* domain. How can the E33A mutation eliminate the action of the Aha1p *C* domain only in *cis* to the Aha1p *N* domain interaction with the Hsp82p M domain (Fig. 8A)?

We propose a model for Aha1p-mediated ATPase stimulation that involves three different steps (Fig. 8B). Interaction of the Aha1p *N* domain with the M domain of one subunit of the Hsp90 dimer drives two different conformational events. The first results in weak ATPase stimulation of the dimer regardless of what subunit is capable of hydrolyzing ATP. Hch1p and the Aha1p *N* domain are capable of driving this first event in an asymmetric manner, that is, from either subunit, as both stimulate the ATPase activity of a heterodimer harbouring one E33A mutation, regardless of which protomer the V391E mutation resides on. The second conformational change does not result in further ATPase stimulation, but induces a conformational change in the *cis* N domain that is critical for the action of the Aha1p *C* domain. This second event ultimately drives a final rearrangement of both *N* domains that allows Aha1p^C^ to exert its effect. In this third step, Aha1p^C^ interacts with the rearranged Hsp90 *N* domains to further simulate ATPase activity.

The precise structural basis for each step of ATPase stimulation is currently unknown as a result of a lack of structural data for the Aha1p-Hsp82p complex. Typically, structural inferences are derived from the structure of the Hsp82p-Sba1p complex^32^,^33^,^45^. However, it is important to note that this structure is an ATPase-inhibited conformation of Hsp82p. Thus, it is challenging to propose a structural model for Aha1p-mediated ATPase stimulation, however, several mechanistic possibilities can be ruled out. We initially hypothesized that Aha1p acts by alleviating lid-mediated inhibition of ATPase activity. However, Aha1p is capable of robustly stimulating Hsp82p:Hsp82p^LL^ heterodimers suggesting that the mechanism for ATPase stimulation is unrelated to alleviation of lid-inhibition. None of the co-chaperone constructs we tested could stimulate ATPase activity when bound to the lidless subunit suggesting that lid deletion blocks the earliest intra-protomer conformational events that drive ATPase stimulation. Moreover, Hch1p inhibited ATPase activity in Hsp82p:Hsp82p^LL^ heterodimers when bound to the lidless subunit. This may result from antagonizing local or global conformational changes that are induced by lid deletion, as a result of the N-M communication (observed by NMR) upon addition of Hch1p. Our analysis also allows us to rule out acceleration of global conformational rearrangements in Hsp90 as the basic mechanism for Aha1p action. Transition between the conformational states associated with the intrinsic ATPase cycle are similarly accelerated upon deletion of one lid segment or the addition of Aha1p^38^. However, both lid deletion and Aha1p binding contribute independently to ATPase stimulation in our experiments. This suggests that the action of Aha1p is at least partly independent of the five global conformational rearrangements identified in previous studies^38^.

A large body of research regarding the structure and function of Hsp90 suggests that it is a highly dynamic, allosteric machine that is regulated by numerous co-chaperones and post-translational modifications (PTMs)^8^,^19^,^28^,^30^,^32^,^33^,^42^,^43^,^44^,^45^,^46^,^47^,^48^,^49^,^50^,^51^,^52^,^53^,^54^,^55^,^56^,^57^. However, a complete view of the catalytic cycle, and mechanism of action for stabilizing client proteins remains elusive. An emerging theme from recent work suggests that the *in vivo* regulation of Hsp90 is dependent on asymmetry in its activation, and has the potential to clarify many aspects underlying the molecular basis of Hsp90 function. A recent example is the observation that Aha1p is recruited to asymmetrically SUMOylated Hsp90^42^. It is interesting that Aha1p^N^ binding to the Hsp82p M domain elicits a significant chemical shift change for K178, the site of SUMOylation. Given the identification of multiple PTMs that are required for Aha1p recruitment to Hsp90, it seems likely that there are numerous species of Hsp90 dimers, characterized by different asymmetric modifications, that may be regulated differently. Two well-characterized sites of phosphorylation within Hsp90 are T22 and Y24^52^,^53^. Similar to SUMOylation of K178, modification of these sites is required for recruitment of Aha1p. In the case of T22, yeast strains that express either non-phosphorylatable (Hsp82p^T22A^) or phospho-mimetic (Hsp82p^T22E^) versions of Hsp82p have similar phenotypes^53^. Moreover, while phosphorylation of T22 is required for Aha1p recruitment, Aha1p is not recovered in complex with either Hsp82p^T22A^ or Hsp82p^T22E^ from yeast lysates. In the case of Y24, a phospho-mimetic version of Hsp82p (Hsp82p^Y24E^) lacks ATPase activity and does not support viability in yeast^52^. A possible explanation for these observations is that T22 and Y24 phosphorylation occurs asymmetrically in a manner similar to that for SUMOylation of K178, raising the possibility that such modifications may enhance or restrict the action of one or both Aha1p domains in a subunit-specific fashion. However, more work is required to understand the relationship between post-translational modifications of Hsp90 and co-chaperone function.

It is important to note that while ATPase activity can be attributed to Hsp82p heterodimers in our assays, measurements regarding the affinity of co-chaperones for these heterodimers are complicated by the presence of the excess, but inactive, pool of Hsp82p homodimers. However, some inferences can be made from the results with the lidless variant. In particular, our experiments with Hsp82p:Hsp82p^LL^ heterodimers (Fig. 2C) stand out from other ATPase assays involving titration of Aha1p. Figure 2C shows a lag in ATPase stimulation, with robust stimulation not occurring until addition of 5 μM Aha1p. This suggests that Aha1p is binding preferentially to the excess, but inactive, pool of Hsp82p^LL^ homodimers present in these experiments. We calculated the apparent affinity of Aha1p for wildtype Hsp82p as ~1.9 μM (Fig. 2D). However, the apparent affinity of Aha1p for the ATPase-competent Hsp82p:Hsp82p^V391E/LL^ heterodimers is ~0.8 μM (Fig. 3E), suggesting that Aha1p has higher affinity for these heterodimers than wildtype Hsp82p homodimers. This is consistent with previous reports showing that Aha1p binds more strongly to *N*-terminally dimerized Hsp82p, which is expected to be caused by lid deletion in one subunit^38^. However, calculation of the exact affinity (in Fig. 3E) is not possible owing to the presence of the excess pool of Hsp82p^V391E/LL^ homodimers. Such calculations would require a different strategy than that employed in the current study.

This study demonstrates the key role of subunit-specific regulation of Hsp90 in the context of co-chaperone regulation. Our model provides a framework for interrogating the effect of post-translational modifications on Aha1p action that can be applied to understand the function of other co-chaperones that interact with the Hsp90 dimer at different sites. Sba1p binds to the dimerized *N* terminal domains of Hsp90 and can inhibit Aha1p-mediated ATPase activity^45^,^58^,^59^,^60^. Sti1p utilizes one of its three tetratricopeptide repeat (TPR) domains to bind to the MEEVD motif at the *C* terminus of Hsp90^61^,^62^. While two Sti1p molecules can bind to the Hsp90 dimer simultaneously, binding to one subunit reduces the affinity at the second site^63^,^64^. This suggests that binding of Sti1p induces asymmetry in the Hsp90 dimer. Asymmetry in binding may also be intrinsic to client:Hsp90 complexes in general. Recent work shows that two molecules of the glucocorticoid hormone receptor (GR) can interact with an Hsp90 dimer, but addition of Sba1p or Sti1p drives formation of an asymmetric complex consisting of only one co-chaperone and one GR^65^.

Functionalization of Hsp90 protomers by asymmetric modification may be a mechanism for co-chaperone recruitment *in vivo*. It remains to be determined whether these modifications occur on one or both subunits, and to what degree they are coordinated. In addition, the nature by which asymmetric modifications alter the function of co-chaperones with respect to client activation and ATPase activity, remains to be resolved. Our three-step model is an important step towards this goal for Aha1p.

## Methods

### Expression plasmid construction

Bacterial expression vectors encoding Hsp82p, Sti1p, Aha1p, Hch1p, Hch1p-Aha1p chimera, and Aha1p^N^ were constructed as previously described (Armstrong 2012, Horvat 2014). The *CPR6* coding sequencing was amplified by PCR with primers designed to introduce *Nde*1 and *Bam*H1 restriction sites at the 5′ and 3′ ends, respectively. The PCR product was digested with *Nde*I and *Bam*HI for ligation into similarly cut pET11dHis. The lidless-mutant of Hsp82 was constructed and kindly provided by Johannes Buchner in a pET28a vector^37^. We digested the lidless Hsp82 (Hsp82p^LL^) construct with *Nde*1 and *Bam*H1 for ligation into similarly cut pET11dHis. Site directed mutagenesis was carried out to construct Hsp82p variants (Hsp82p^V391E^, Hsp82p^D79N^, Hsp82p^E33A^, Hsp82p^LL^, Hsp82p^V391E/LL^, Hsp82p^V391E/D79N^, and Hsp82p^V391E/E33A^) using QuikChange mutagenesis (Agilent). The coding sequences contained in all mutagenized plasmids were verified by sequencing. The myc and the HA epitope was fused in-frame with the 6xHis-tag sequence upstream of the *Nde*I site of the pET11dHis vector. The coding sequences for Aha1p, Hsp82p and Hsp82p^V391E^ were cloned into this pET11dHisMyc vector, and the coding sequences for Hsp82p^D79N^ and Hsp82p^E33A^ were cloned into the pET11dHisHA vector, as described above.

### Protein Expression and Purification

Protein expression and purification was carried out as previously described^28^,^29^. *S. cerevisiae* Hsp82p, Hsp82p variants, and co-chaperones were expressed in *Escherichia coli* strain BL21 (DE3) from pET11dHis or pET11dHisMyc (Stratagene, La Jolla, Califonria, USA). Cells were grown to an OD600 of 0.8–1.0, induced with 1mM isopropyl-1-thio-D-galactopyranoside (IPTG), and then incubated at 37 °C. Cells expressing co-chaperone proteins (Aha1p, Hch1p, and Aha1p^N^, Hch1p-Aha1p chimera) were harvested after 8 hours of growth. Cells expressing Sti1p, Cpr6p, Hsp82p and variants (Hsp82p^V391E^, Hsp82p^D79N^, Hsp82p^E33A^, Hsp82p^LL^, Hsp82p^V391E/LL^, Hsp82p^V391E/D79N^, and Hsp82p^V391E/E33A^) were harvested after overnight growth. Cells were harvested by centrifugation and stored at −80 °C.

Bacterial pellets were resuspended in lysis buffer (25 mM NaH_2_PO_4_ pH 7.2, 500 mM NaCl, 1 mM MgCl_2_, 20 mM Imidazole, 5 mM β-mercaptoethanol), supplemented with HALT EDTA-free protease inhibitor (Thermoscientific), and lysed using Avestin Emulsiflex C3 (Avestin, Ottawa, Ontario, Canada). Lysates were clarified by ultracentrifugation at 36,000 rpm for 30 minutes and His-tagged proteins were isolated on a HisTrap FF column using an AKTA Explorer FPLC (GE Healthcare). Isolated 6xHis-tagged co-chaperone containing protein fractions were pooled and concentrated and further purified by size exclusion chromatography on a Superdex 75 column (GE Healthcare) in 25 mM Hepes, pH 7.2, 50 mM NaCl, 5 mM ß-mercaptoethanol. Isolated 6xHis-tagged Hsp82p containing protein fractions were pooled and concentrated in the presence of 5 mM EDTA, and then further purified by size exclusion chromatography on a Superdex 200 (GE Healthcare) in 25 mM Hepes pH 7.2, 10 mM NaCl, 5 mM ß-mercaptoethanol.

For NMR studies, ^2^H,^15^N-Hsp82p N-M domain (Hsp82p^NM^), as well as unlabeled Aha1p *N*-domain (Aha1p^N^), full-length Aha1p, and Hch1p were produced for high-resolution nuclear magnetic resonance (NMR) spectroscopy studies. Aha1p^N^ and Aha1p were expressed and purified as described above. Hch1p was expressed in 2×TY media. Protein expression was induced at an OD_600_ ~0.6 with 0.4 mM IPTG and incubation overnight at 25 °C. ^2^H,^15^N-Hsp82p^NM^ was expressed using the method of Marley *et al*.^66^; *E. coli* were grown in 2 L of LB media to an OD_600_ ~0.6, pelleted, and transferred to 0.5 L of M9 minimal media containing 99% D_2_O, 0.5 g (^15^NH_4_)_2_SO_4_ and 4 g unlabeled glucose. The cells were incubated for 2 hr, subsequently induced with 0.4 mM IPTG, and grown overnight at 25 °C. Cells expressing Hch1p and Hsp82p^NM^ were lysed by sonication and purified using FPLC with HisPrep 16/10 and Superdex75 26/60 columns.

### ATPase Assays

ATPase assays were carried out using the enzyme coupled assay as previously described where the regeneration of ATP is coupled to the oxidation of NADH^27^,^28^,^29^,^67^. All reactions were carried out in triplicate, three times in 100 μL volumes using a 96-well plate. Activity was detected as a decrease in absorbance at 340 nm which was measured every minute for 90 minutes using a BioTek Synergy 4 and the path-length correction function. Average values of the experiments are shown with error expressed as standard error of the mean. The decrease in NADH absorbance at 340 nm was converted to micromoles of ATP using Beer’s Law and then expressed as a function of time^68^. The final conditions of all the reactions are 25 mM Hepes (pH 7.2), between 1–25mM NaCl, 5 mM MgCl_2_, 1 mM DTT, 0.6 mM NADH, 2 mM ATP, 1 mM phosphoenol pyruvate (PEP), 2.5 μL of pyruvate kinase/lactate dehydrogenase (PK/LDH) (Sigma), and 0.5% DMSO. Identical reactions were quenched with 100 μM NVP-AUY922 and subtracted from unquenched reactions to correct for contaminating ATPase activity. All ATPase assays were carried out at 30 °C with recombinant proteins harboring *N*-terminal 6xHis tags.

Heterodimer ATPase Assays were performed as described earlier in Retzlaf *et al*.^33^. We allowed equilibration of heterodimers by mixing two samples of Hsp82p (one being a functional, ATPase-competent Hsp82p and the other, an ATPase-dead Hsp82p variant) at a specific concentration ratio for 15 minutes at room temperature. In Figs 2 and 3, Hsp82p heterodimers were formed by mixing functional, ATPase competent Hsp82p (wildtype Hsp82p and Hsp82p^V391E^) with the ATPase dead Hsp82p variant (Hsp82p^LL^ or Hsp82p^V391E/LL^). Hsp82p^LL^ was titrated into 2 μM wildtype Hsp82p or Hsp82p^V391E^ and Hsp82p^V391E/LL^ was titrated into 2 μM Hsp82p to determine at which ratio we have to mix the ATPase competent Hsp82p with the ATPase dead Hsp82p to ensure >80% heterodimer formation (based on resulting ATPase rate). We determined that a 2:10 ratio (or 1:5 ratio of ATPase competent to ATPase dead) results in >80% heterodimer formation, and used this 1:5 ratio in the following co-chaperone titration experiments. Heterodimers were first formed by incubating them separately for 15 minutes prior to adding them to specific wells. Co-chaperones were then added to the wells containing the heterodimer mix. In Figs 4 and 5, the ATPase-dead variants (Hsp82p^E33A^, Hsp82p^D79N^, Hsp82p^V391E/E33A^ or Hsp82p^V391E/D79N^) were titrated into reactions containing functional, ATPase-competent, Hsp82p (wildtype Hsp82p or Hsp82p^V391E^) and a defined concentration of co-chaperone. First, the ATPase competent Hsp82p was added to wells, followed by the addition of the ATPase-dead variants of a specific concentration. We allowed equilibration of heterodimers to occur by waiting 15 minutes, and then added the co-chaperones to the wells.

For the Sti1p displacement ATPase assay in Fig. 7, 4 μM Aha1p, Chimera, Hch1p, or Aha1p^N^ were added to the designated wells first, followed by 4 μM Sti1p and Cpr6p. Lastly, 2 μM wildtype Hsp82p was added to these reactions. Statistical significance was measured using one way ANOVA analysis.

All ATPase assay was started by the addition of the regenerating system consisting of MgCl_2_, DTT, NADH, ATP, PEP, and PK/LDH. Fit lines were calculated according to the following equation (Y = ((Bmax*X)/(Kapp + X)) + X_0_)^33^. The ATPase rates are either shown as μM ATP hydrolyzed per minute per μM of Hsp82p (1/min) or as a fold ATPase stimulated rate of the starting Hsp82p or heterodimer intrinsic rate.

V_max_ (Fig. 2D) was calculated using the Michaelis-Menten non-linear fit in GraphPad Prism.

### *In vitro* immunoprecipitation

*In vitro* immunoprecipitation assays were conducted using Ultralink Protein G beads (Pierce Thermo Fisher) that had been coupled to anti-myc monoclonal antibodies (clone 9E10) at a concentration of 5 μg antibody per 1 μL of beads. All recombinant proteins used in these assays harbour *N*-terminal 6xHis tags, and where specified, the additional myc-tag (6xHis-myc) or HA-tag (6xHis-HA).

To assess Aha1p binding, 5 μM Hsp82p (wildtype or mutant – Hsp82p^V391E^, Hsp82p^D79N^, Hsp82p^E33A^, Hsp82p^LL^) was mixed with 5 μM 6xHis-myc-tagged Aha1p. To assess Sti1p displacement from Hsp82p, 5 μM Hsp82p was incubated with 5 μM 6xHis-myc-tagged Sti1p in the presence of 5 μM Cpr6p, with and without 10 μM Aha1p or 50 μM of Aha1p^N^, and 5 mM AMPPNP. Band intensities of Hsp82p and myc-Sti1p from Coomassie stained gels were measured using the multiplex band analysis function of AlphaView software (FluorochemQ, Protein Simple). The ratio of Hsp82p to myc-Sti1p was used to measure the efficiency of recovery in each condition. The percent displacement represents the average relative reduction in this ratio from three independent experiments. Statistical significance was measured using one way ANOVA analysis.

To assess heterodimer formation, 5 μM 6xHis-myc-tagged Hsp82p or Hsp82p^V391E^ was incubated with 5 μM 6xHis-HA-tagged Hsp82p^D79N^ or Hsp82p^E33A^ for 15 minutes. We added 10 μL of Ultralink Protein G beads coupled to anti-myc monoclonal antibodies to all these 50 μL reactions and then incubated them on a rotator at room temperature for 90 minutes. Beads were then pelleted, washed once in 250 μL of binding buffer, resuspended in 50 μl SDS sample buffer, and run on SDS-PAGE. Complexes were analyzed by Coomassie blue staining or western blotting. Myc-tagged proteins were detected with mouse anti-myc monoclonal antibody (4A6, Millipore), His-tagged proteins were detected with mouse anti-Tetra-His monoclonal antibody (34670, Qiagen), and HA-tagged proteins were detected with rat anti-HA monoclonal antibody (3F10, Roche). Final buffer conditions of all reactions were 25 mM Hepes (pH 7.2), 10–15 mM NaCl, 5 mM MgCl_2,_ and 0.1% tween-20.

### NMR spectroscopy

BCA analysis was used to determine the concentration of purified proteins. Samples for NMR contained ~0.25 mM ^2^H,^15^N-Hsp90-NM, 20 mM sodium phosphate, 50 mM NaCl, 5% D_2_O, 1 mM 4,4-dimethyl-4-silapentane-1-sulfonic acid as a chemical shift reference, pH 7.5. Samples containing co-chaperone included 0.5 mM unlabeled Aha1p^N^ or Hch1p. Samples containing ATP included 2 mM ATP and 4 mM MgCl_2_. 2D ^1^H-^15^N TROSY-HSQC NMR spectra were acquired on a 600 MHz spectrometer at 25 °C. Spectra were processed using NMRPipe^69^ and analyzed using NMRViewJ^70^. The spectrum of Hsp82p^NM^ in the free state was assigned using chemical shifts deposited in the Biological Magnetic Resonance Bank database for the yeast Hsp90-N^71^ and Hsp90-M^72^ domains. Peaks in the spectra of Hsp82p^NM^ bound to co-chaperone and/or ATP were assigned mainly based on the closest peak to the free-state spectrum^40^. Chemical shift changes were calculated using Δδ = [(Δδ^15^N/5)^2^ + (Δδ^1^H^N^)^2^]^1/2^.

## Additional Information

**How to cite this article**: Wolmarans, A. *et al*. The Mechanism of Hsp90 ATPase Stimulation by Aha1. *Sci. Rep.* **6**, 33179; doi: 10.1038/srep33179 (2016).

## Supplementary Material

Supplementary Information

## Figures and Tables

**Figure 1:**
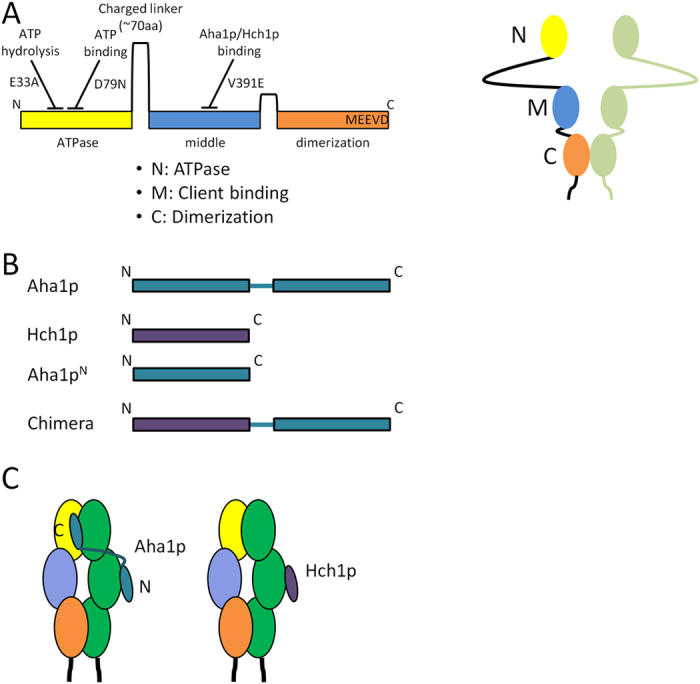
Structure and interaction of Hsp90 with Aha1 and Hch1. (**A**) Hsp90 is comprised of three domains; an *N* terminal ATPase domain (yellow), a middle domain (blue), and *C* terminal dimerization domain (orange). Each domain is joined by a charged linker and the last five residues (MEEVD) comprise a docking site for a class of co-chaperones characterized by a tetratricopeptide repeat domain. (**B**) Aha1p is an Hsp90 co-chaperone comprised of two domains; an *N* terminal domain and a *C* terminal domain. Hch1p is a homologue of Aha1p but corresponds to only the Aha1p *N* domain. Also used in this study are the individual *N* domain of Aha1p (Aha1p^N^) and a chimera comprised of Hch1p fused to the *C* domain of Aha1p (Chimera). (**C**) The Aha1p *N* domain and Hch1p interact with the Hsp90 middle domain and the Aha1p *C* domain interacts with the dimerized *N* terminal domains of Hsp90.
