# Supplementary material for: The Mechanism of Hsp90 ATPase Stimulation by Aha1
Source: Sci Rep. 2016 Sep 12;6:33179. doi: 10.1038/srep33179 (PMC5018835; doi:10.1038/srep33179)
Supplement: Supplementary Information [file srep33179-s1.pdf]

The Mechanism of Hsp90 ATPase Stimulation by Aha1

Annemarie Wolmarans[1], Brian Lee[2], Leo Spyracopoulos[2], and Paul LaPointe[1*]

From the Departments of Cell Biology[1] and Biochemistry[2], University of Alberta, Edmonton, Alberta, CANADA, T6G 2H7

* - Corresponding author (paul.lapointe@ualberta.ca)

Supplemental Figures:

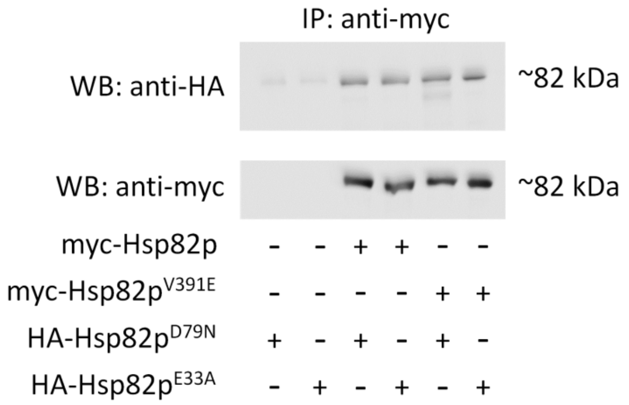

Figure S1: Heterodimers formation between wildtype Hsp82p, Hsp82p^{V391E}, Hsp82p^{D79N} and Hsp82p^{E33A}. Hsp82p and Hsp82p^{V391E} both form heterodimers with Hsp82p^{D79N} and Hsp82p^{E33A} as HA-tagged Hsp82p^{D79N} and Hsp82p^{E33A} co-ip with Myc myc-Hsp82p and myc-Hsp82p^{V391E} pull down Hsp82p^{D79N} and Hsp82p^{E33A}. 5uM purified His-Myc-tagged Hsp82p or Hsp82p^{V391E} was incubated with 5uM purified His-Flag-tagged Hsp82p^{D79N} or Hsp82p^{E33A} for 15 minutes. These reactions were incubated on a rotator at room temperature for 90 min. Beads were pelleted, washed once in 250uL of binding buffer, run on SDS-PAGE, and analyzed by western.

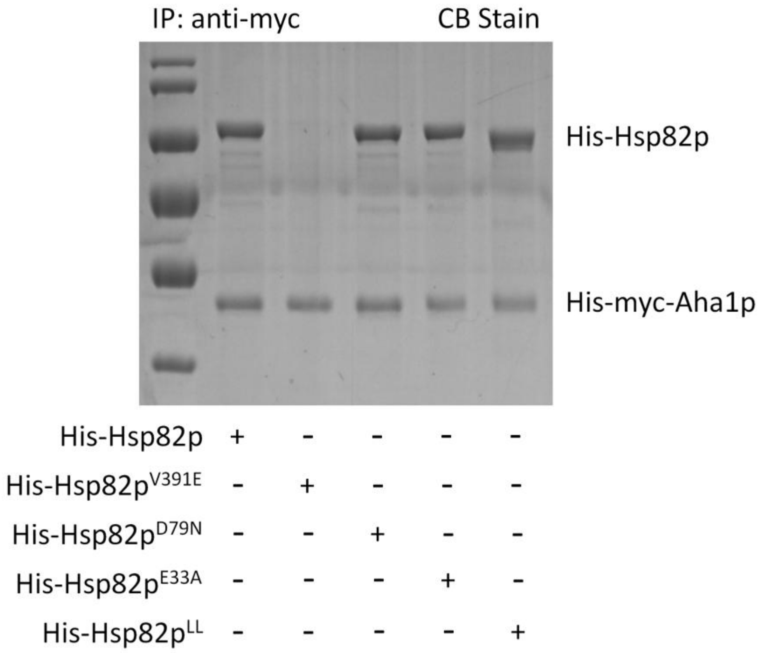

Figure S2: Hsp82p variants bind Aha1p. Wildtype Hsp82p, Hsp82p^{D79N}, Hsp82p^{E33A}, and Hsp82p^{LL} all form a stable complex with myc-tagged Aha1p *in vitro*. Hsp82p^{V391E} harbours a mutation that prevents the formation of a stable complex with Aha1p. 5uM of Hsp82 variants were incubated with 5uM 6x his-myc-tagged Aha1p. Complexes were isolated with beads coupled to anti-myc monoclonal antibody 9E10, run on SDS-PAGE and analyzed by Coomassie blue staining (CB).

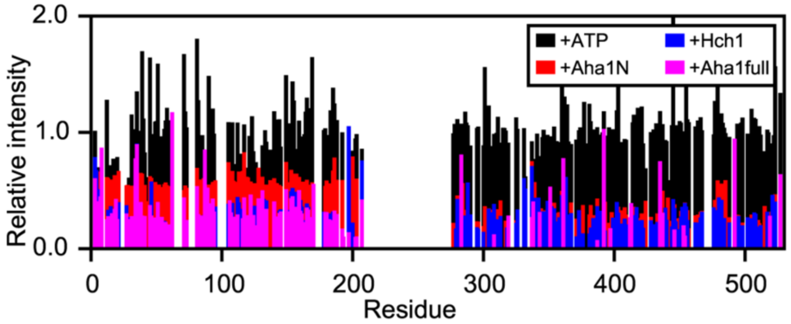

Figure S3. Chemical shift and peak intensity analysis in an Hsp82p N-M construct upon ATP, and co-chaperone binding. Peak intensity changes in Hsp82p N-M construct NMR spectra upon addition of ATP (black), Aha1p^{N} (red), Hch1p (blue), and Aha1p (pink).
